# Clinical and Histopathological Correlations With Blood Groups in Patients With Gastric Cancer

**DOI:** 10.7759/cureus.91161

**Published:** 2025-08-28

**Authors:** Güner Kilic, Fatma Yilmaz, Nukhet Bayram Kayar, Ozan Durmaz, Adnan Ozkahraman, Sevki Konur, Ramazan Dertli, Yusuf Kayar

**Affiliations:** 1 Gastroenterology, Etlik City Hospital, Ankara, TUR; 2 Hematology, Etlik City Hospital, Ankara, TUR; 3 Family Medicine, Van Provincial Health Directorate, Van, TUR; 4 Internal Medicine, Van Training and Research Hospital, Van, TUR; 5 Gastroenterology, Duzce University Faculty of Medicine, Düzce, TUR; 6 Gastroenterology, Necmettin Erbakan University, Konya, TUR; 7 Gastroenterology, Van Education and Research Hospital, Van, TUR

**Keywords:** bloods groups, cancer, clinical, correlations, histopathological, stomach

## Abstract

Introduction

Gastric cancer results from intricate gene-environment interactions, and defining both modifiable and inherited risk factors could improve prevention and personalised therapy. Although ABO blood groups have been linked to gastric cancer susceptibility, findings are inconsistent.

Materials and methods

We retrospectively compared 214 histologically confirmed gastric cancer cases with 214 age- and sex-matched healthy controls. Presenting symptoms, physical findings (palpable mass, jaundice, ascites, hepatomegaly) and upper-gastrointestinal bleeding were recorded. Endoscopy documented tumour location (cardia, corpus, antrum, incisura angularis, whole stomach) and Borrmann type. Biopsies were classified as adenocarcinoma, signet-ring cell carcinoma, neuroendocrine tumour or lymphoma. ABO blood group was determined serologically, and tumour-node-metastasis (TNM) stage assigned by imaging.

Results

The cancer cohort comprised 149 men and 65 women and the control group 107 men and 107 women. ABO distributions were as follows: A, 50.5 %; B, 16.8 %; AB, 2.8% and O, 29.9 % in cases, versus 43.0%, 16.4%, 8.0% and 32.6% in controls, respectively. Blood group A showed a non-significant excess risk, whereas AB showed a non-significant deficit (p>0.05). Epigastric pain occurred significantly more often in AB and O groups than in A and B groups (p<0.05). No other clinical, endoscopic, histological or staging variables differed by blood group.

Conclusions

Our findings suggest a potential increase in gastric cancer risk associated with blood group A, and a possible protective effect of group AB, although statistical significance was not achieved. These observations underscore the need for larger, multicentre prospective studies incorporating genetic and environmental factors to clarify the mechanistic role of ABO antigens in gastric carcinogenesis and to improve risk stratification and early detection strategies.

## Introduction

Stomach cancer is a global health problem, ranking as the fifth most frequently diagnosed malignancy worldwide and the third leading cause of cancer-related death, following lung and colorectal cancers [1]. The incidence of stomach cancer increases with advancing age and reaches a plateau between 55 and 80 years. It is generally seen less in adults below the age of 50 years, and is two- to three-fold more common in men than women [2,3]. The rate of early diagnosis of stomach cancer is low. In many patients, the disease is at an advanced stage at the time of diagnosis, and therefore the optimal time for treatment has passed [4].

The emergence and development of stomach cancer is due to the interaction of genetic and environmental factors. Understanding these risk factors can provide better preventative measures for patients and the development of more rational treatment options [5]. ABO blood grouping is one of the genetic factors, which was first described by Karl Landsteiner in 1901 [6]. ABO blood types are defined by carbohydrate particles bound to a protein known as H antigen on the surface of red blood cells (RBCs). ABO antigens are also highly expressed on epithelial surfaces of the gastrointestinal, bronchopulmonary, and urogenital tracts. Alterations in surface glycoconjugates may affect membrane signaling, intercellular adhesion, and immune function [7,8].

The role of ABO blood groups in stomach cancer was first suggested by Aird et al. in 1953, with the report that blood group A was determined more in stomach cancer patients than in control subjects [9]. Since that time, the relationship between ABO blood groups and the risk of stomach cancer has been comprehensively investigated, but findings have remained inconclusive or inconsistent across studies. The aim of this study was to demonstrate the relationship between the blood groups of patients diagnosed with stomach cancer and their clinical, endoscopic, and histopathological findings.

## Materials and methods

Patient and control group selection

This retrospective study included 214 patients who were diagnosed with stomach cancer as a result of upper gastrointestinal (GI) endoscopy performed in the Endoscopy Unit of the Gastroenterology and Hepatology Department of our hospital between October 2016 and March 2023. A healthy control group was formed of 214 subjects who presented at the Gastroenterology Polyclinic in the same time period and had no pathological findings. The patients included were those aged >18 years who provided written informed consent for participation in the study. Patients were excluded from the study if they were lost to follow-up, if their data were not available or were incomplete, or if they did not wish to participate despite meeting the study inclusion criteria. All the participants were evaluated by a gastroenterologist. After the anamnesis was taken, each patient underwent a physical examination. Routine laboratory investigations included fasting blood glucose, urea, creatinine, aspartate aminotransferase (AST), alkaline phosphatase (ALP), gamma-glutamyl transferase, (GGT), total bilirubin, albumin, total cholesterol, low-density lipoprotein (LDL), triglycerides, prothrombin time, hemogram, thyroid-stimulating hormone (TSH), glycated haemoglobin (HbA1c), C-reactive protein (CRP), sedimentation rate, blood type, fecal occult blood, microscopic and parasite examinations and *Helicobacter pylori* antigen in faeces.

For all the patients and control group subjects, the demographic and anthropometric characteristics (age, gender, body mass index (BMI)) were documented. BMI was calculated as weight in kilograms divided by height in meters squared (kg/m²). Blood group analysis was performed in all the study subjects.

Patients diagnosed with gastric cancer

Patients with a histologically confirmed diagnosis of gastric cancer following upper gastrointestinal endoscopy were included. Clinical symptoms at presentation were recorded, including epigastric pain, chest pain, bloating, nausea and vomiting, dysphagia, feeling of obstruction, weight loss, and reflux symptoms was recorded. In the physical examination, the presence of a palpable mass, jaundice, ascites, and hepatomegaly was investigated and patients who presented with upper gastrointestinal bleeding were also identified. In the upper GI endoscopy, detailed notes were taken of the tumour localisation (cardia, corpus, antrum, incisura angularis, whole stomach) and tumour type according to the Borrman classification (polypoid, ulcerated, ulceroinfiltrative, infiltrative). Pathologies taken from the stomach were stained with hematoxylin and eosin and evaluated by experienced pathologists. Biopsy samples were evaluated histopathologically and categorised as adenocarcinoma, signet ring cell, neuroendocrine, and lymphoma. The blood groups of the patients were examined. Abdominal and thorax computed tomography (CT) and positron emission computed tomography (PET-CT) scans were taken, and tumour-node-metastasis (TNM) grading was performed.

Ethics statement

Written informed consent for participation in the research was provided by all the study participants. The study was approved by the institutional ethics committee (approval number 60KAEK-2025-03-01) and conducted in accordance with the Helsinki Declaration.

Statistical analysis

The data obtained in the study were analyzed statistically using the Statistical Package for the Social Sciences version 22.0 (IBM Corp., Armonk, NY, USA). Continuous variables were presented as mean±standard deviation (SD) values, and categorical variables as frequency and percentage (n (%)). Data were tested for compliance with normal distribution using the Kolmogorov-Smirnov test and histogram inspection. Parametric data of the groups were compared using the Student’s t-test and the one-sample chi-square or two-proportions Z test was used to test categorical data. In comparison with fewer than five observations, Fisher’s exact test was applied, and some data were analyzed using one-way analysis of variance (ANOVA) post hoc (least significant difference (LSD)) test. A value of p<0.05 was considered statistically significant.

## Results

A total of 428 individuals were evaluated, comprising 214 patients (aged 25-84 years) diagnosed with gastric cancer and 214 healthy controls (aged 18-82 years). The gastric cancer group included 149 (69.6%) men and 65 (30.4%) women, whereas the control group included 107 (50.0%) men and 107 (50.0%) women. The gender distribution differed significantly between the groups (p<0.001).

In the gastric cancer group, the mean height was 165.9±8.3 cm (range: 150-186 cm), mean weight was 71.7±12.5 kg (range: 42-98 kg), and mean body mass index (BMI) was 26.05±4.3 kg/m² (range: 16.4-37.7). In the control group, the mean height was 165.1±9.1 cm (range: 151-187 cm), mean weight was 75.6±15.2 kg (range: 49-125 kg), and mean BMI was 27.9±6.3 kg/m² (range: 23.1-41.1). There was no statistically significant difference in height between the groups (p>0.05), whereas both weight and BMI were significantly lower in the gastric cancer group (p=0.001 and p<0.001, respectively).

In the gastric cancer group, blood group distribution was as follows: A in 108 patients (50.5%), B in 36 (16.8%), AB in six (2.8%), and O in 64 (29.9%). In the control group, the distribution was A in 92 individuals (43.0%), B in 35 (16.4%), AB in 17 (8.0%), and O in 70 (32.6%). Although blood group A appeared more prevalent and AB less prevalent among cancer patients, the differences were not statistically significant (p>0.05) (Table 1).

**Table 1:** Demographic, anthropometric and blood group characteristics in gastric cancer and control groups

	Gastric cancer	Control	Test Statistic	P value
	n=214	n=214		
Age	62.5±13.1	47.6±14.9	t = 2.48	0.013
SD, min-max	(25-84)	(18-82)		
Gender			χ² = 15.63	<0.001
Female	65 (30.4%)	107 (50%)		
Male	149 (69.6%)	107 (50%)		
Size	165.9±8.3	165.1±9.1	t = 0.34	0.738
Min-max	(150-186)	(151-187)		
Weight	71.7±12.5	75.6±15.2	t = 3.29	0.001
Min-max	(42-98)	(49-125)		
BMI	26.05±4.3	27.9±6.3	t = 4.19	<0.001
Min-max	(16.4-37.7)	(23.1-41.1)		
Blood Group			χ² = 6.81	0.078
A	108 (50.5%)	92 (43.0%)		
B	36 (16.8%)	35 (16.4%)		
AB	6 (2.8%)	17 (8.0%)		
O	64 (29.9%)	70 (32.6%)		
SD: Standard deviation, Min: Minimum, Max: Maximum				

The association between ABO blood group and various clinical, endoscopic and histopathological characteristics was further analyzed. Epigastric pain occurred significantly more frequently in patients with blood groups AB and O compared to those with groups A and B. Chest pain was more commonly reported in group AB, while bloating was more frequent in group O than in group A. No significant differences were noted among the blood groups in terms of palpable mass, weight loss, ascites, hepatomegaly, thrombosis, or nausea. Vomiting was significantly more common in groups O and B than in group A. Dysphagia and a sensation of obstruction were less frequent in group AB. Gastrointestinal bleeding and jaundice were more often reported in groups A and B, while reflux symptoms occurred more commonly in groups B and O (p<0.05) (Table 2).

**Table 2:** The relationship between clinical and tumor characteristics and blood type in patients with gastric cancer

	A (n=108)	B (n=36)	AB (n=6)	0 (n=64)	Test Statistic (χ²)	P value
Clinical features						
Epigastric Pain	61 (56.5%)	16 (44.4%)	6 (100%)	60 (93.8%)	16.21	<0.001
Chest Pain	43 (39.8%)	17 (47.2%)	6 (100%)	34 (53.1%)	9.87	0.019
Bloating	40 (37.0%)	19 (52.8%)	3 (50.0%)	40 (62.5%)	10.36	0.012
Palpable Mass	8 (7.4%)	2 (5.6%)	0 (0.0%)	4 (6.3%)	0.58	0.892
Nausea	66 (61.1%)	25 (69.4%)	3 (50.0%)	45 (70.3%)	2.40	0.491
Vomiting	34 (31.5%)	21 (58.3%)	3 (50.0%)	38 (59.4%)	13.34	0.001
Dysphagia	57 (52.8%)	22 (61.1%)	0 (0.0%)	27 (42.2%)	9.64	0.022
Stuck Feeling	61 (56.5%)	22 (61.1%)	0 (0.0%)	31 (48.4%)	8.79	0.032
GI bleeding	20 (18.5%)	7 (19.4%)	0 (0.0%)	4 (6.3%)	6,81	0.084
Weight Loss	98 (90.7%)	33 (91.7%)	6 (100%)	61 (95.3%)	1,72	0.628
Jaundice	12 (11.1%)	8 (22.2%)	0 (0.0%)	0 (0.0%)	11,42	0.002
Ascites	4. (3.7%)	4 (11.1%)	0 (0.0%)	4 (6.3%)	3,22	0.361
Hepatomegaly	8 (7.4%)	0 (0.0%)	0 (0.0%)	4 (6.3%)	3,22	0.361
Thrombosis	2 (1.9%)	1 (2.8%)	0 (0.0%)	1 (1.6%)	0,18	0.958
Reflux Symptoms	69 (63.9%)	29 (80.6%)	3 (50.0%)	60 (93.8%)	17,33	<0.001
Stomach placement - Cardia						
Corpus	65 (60.2%)	25 (69.4%)	3 (50.0%)	16 (25.2%)	16.21	<0.001
Antrum	4 (3.7%)	0 (0.0%)	0 (0.0%)	0 (0.0%)		
Incisura angularis	7 (6.5%)	7 (19.4%)	0 (0.0%)	9 (14.1%)		
Whole stomach	20 (18.5%)	0 (0.0%)	3 (50.0%)	27 (42.2%)		
	12 (11.1%)	4 (11.1%)	0 (0.0%)	12 (18.8%)		
Borman classification					9.87	0.004
Polypoid	6 (5.6%)	3 (8.3%)	0 (0.0%)	0 (0.0%)		
Ulceration	25 (23.1%)	4 (11.1%)	3 (50%)	27 (42.2%)		
Ulseroinfiltrative	39 (36.1%)	21 (58.3%)	3 (50%)	19 (29.7%)		
Infiltrative	38 (35.2%)	8 (22.2%)	0 (0.0%)	18 (28.1%)		
Histopathology					10.36	<0.001
Adenocarcinoma	85 (78.7%)	36 (100%)	6 (100%)	45 (70.3%)		
Signet-ring cell	11 (10.2%)	0 (0.0%)	0 (0.0%)	8 (12.5%)		
Neuroendocrine	8 (7.4%)	0 (0.0%)	0 (0.0%)	0 (0.0%)		
Lymphoma	4 (3.7%)	0 (0.0%)	0 (0.0%)	11 (17.2%)		
T Staging					6.44	0.174
Under T4	21 (19.4%)	3 (8.3%)	0 (0.0%)	15 (23.4%)		
T4	87 (80.6%)	33 (91.7%)	6 (100%)	49 (76.6%)		
N Staging					5.97	0.051
N1	38 (35.2%)	4 (11.1%)	3 (50.0%)	26 (40.6%)		
N2	48 (44.4%)	24 (66.7%)	2 (33.3%)	26 (40.6%)		
N3	22 (20.4%)	8 (22.2%)	1 (16.7%)	12 (18.8%)		
M Staging					3.26	0.198
M0	69 (63.99%)	20 (55.6%)	6 (100%)	38 (59.4%)		
M1	39 (36.1%)	16 (44.4%)	0 (0.0%)	26 (40.6%)		

With respect to tumor localization, cardia involvement was significantly more frequent in blood groups A and B compared to group O. Tumors localized in the antrum were more common in group B than in groups A and AB, whereas tumors in the incisura angularis were more frequent in groups AB and O than in A and B. No significant differences were found between the groups regarding involvement of the corpus or the whole stomach (Table 2).

Regarding endoscopic findings, polypoid tumors were significantly more frequent in group B compared to groups A and O, and more frequent in group A than in group O. No significant differences were observed among the blood groups in the ulcerated, infiltrative, or ulceroinfiltrative tumor types (Table 2).

Histopathological evaluation revealed that adenocarcinoma was significantly more common in blood groups B and AB, whereas signet-ring cell carcinoma was more prevalent in groups A and O. Neuroendocrine tumors were more frequently observed in group A, and lymphoma was more common in group O (p<0.05) (Table 2). There were no statistically significant differences in T or M staging between blood groups. Although not statistically significant, the frequency of N1-stage disease was lower and N2-stage disease was higher in group B compared to the other groups (Table 2).

## Discussion

The association between various cancer types and ABO blood groups has been explored extensively over the years [10-12]. Since the seminal study by Aird et al., which reported a link between ABO blood types and both peptic ulcer and gastric carcinoma, numerous investigations have been published. Several of these studies have suggested that individuals with blood group A may carry an approximately 20% increased risk of developing gastric cancer [13,14]. In the present study, consistent with earlier findings, blood group A was more frequently observed among gastric cancer patients, while blood group AB appeared to be underrepresented. This latter finding aligns with a previous Turkish study by Tuncel et al., which similarly reported a lower incidence of gastric cancer in individuals with blood group AB [15].

However, the literature remains inconsistent. In a large cohort study involving 4,932 gastric cancer patients, Mao et al. found an increased risk not only in individuals with blood group A, but also in those with blood group AB. These discrepancies may be attributable to geographic variations, differences in environmental exposures, or population-specific genetic backgrounds. While a consistent association has been observed between blood group A and gastric cancer across multiple studies, fewer have reported a significant relationship between blood group AB and cancer risk [16].

Several hypotheses have been proposed to explain the biological mechanisms linking ABO blood types and gastric cancer. These include roles in systemic inflammation, cell adhesion, and membrane signaling alterations. A potential link between ABO blood type and *Helicobacter pylori* infection has also been postulated, particularly in relation to cagA-positive strains that provoke heightened inflammatory responses [17]. A meta-analysis demonstrated that individuals with blood group A have increased susceptibility to *H. pylori* infection compared to those with other ABO blood types, potentially contributing to carcinogenesis [18].

Regarding prognosis, the impact of ABO blood group remains controversial. Qiu et al. reported no prognostic relevance of blood type in a cohort of 474 gastric cancer patients [19]. Conversely, Xu et al., in a retrospective analysis of 1,412 patients, found that blood group AB was associated with better prognosis [20]. In the current study, no significant associations were observed between ABO blood groups and advanced clinical features such as palpable mass, weight loss, ascites, hepatomegaly, or thrombosis. This suggests that blood type may not correlate with disease severity or stage at presentation.

There is limited evidence on the association of ABO blood groups with demographic and tumor characteristics such as age, sex, tumor location, size, endoscopic appearance, T stage, and lymph node involvement. In this study, tumor localization was most frequently in the cardia (50.9%), followed by the incisura angularis (23.4%). Cardia involvement was more common in patients with blood groups A and B, whereas tumors in the incisura angularis were more frequently associated with blood groups AB and O. Histopathological evaluation revealed adenocarcinoma in 80.4% of patients, predominantly among those with blood groups B and AB. Although TNM staging did not show statistically significant differences among blood groups, N2 lymph node involvement appeared more frequent in patients with blood group B. In contrast, a study by Yu et al. involving 897 gastric cancer patients found no significant association between ABO blood groups and clinical parameters, including age, sex, tumor site, size, histological differentiation, T stage, or lymph node metastasis [21].

This study has several limitations. Primarily, it was a single-center, retrospective study with a relatively modest sample size, which may limit the generalizability of the findings. The study also did not make statistical adjustments for known risk factors for stomach cancer, such as *H. pylori* infection, smoking, alcohol consumption, diet, and socioeconomic status. Nonetheless, a notable strength of the study is the comprehensive evaluation of ABO blood group in relation to clinical, endoscopic, histological, and prognostic features in gastric cancer patients.

## Conclusions

The results of this study revealed significant associations between ABO blood groups and various clinical and pathological features of gastric cancer, including disease risk, tumor localization, and histological subtype. Specifically, individuals with blood group A showed a higher prevalence of gastric cancer, while those with blood group AB had a lower frequency. Furthermore, blood group distribution appeared to correlate with certain symptoms at presentation, endoscopic tumor localization, and histopathological classifications, such as adenocarcinoma and signet ring cell carcinoma. These findings suggest that blood group antigens may play a role not only in disease susceptibility but also in tumor behavior and biological diversity. Given the retrospective and single-center nature of this study, future large-scale, multicenter, and prospective studies are warranted to validate these associations and explore their potential impact on disease prognosis, treatment strategies, and personalized medicine approaches in gastric cancer management.
